# Medroxyprogesterone Acetate versus Gonadotropin-Releasing Hormone Antagonist for the Prevention of Premature Luteinizing Hormone Surge in hyper-responder women undergoing controlled ovarian stimulation for IVF/ICSI Cycles

**DOI:** 10.5935/1518-0557.20220006

**Published:** 2023

**Authors:** Sunita Tandulwadkar, Shruti Gupta, Akhileshwar Singh, Sneha Mishra, Samta Singhania

**Affiliations:** 1 Ruby Hall Clinic, Pune, India

**Keywords:** medroxyprogesterone acetate, premature LH surge, GnRH antagonist, controlled ovarian stimulation, hyper-responders

## Abstract

**Objective:**

To compare the effect of Medroxyprogesterone acetate versus Gonadotropin releasing hormone antagonist for the prevention of premature luteinizing hormone (LH) surge in infertile hyper-responder women undergoing controlled ovarian stimulation for in vitro fertilization (IVF) /intracytoplasmic sperm injection (ICSI) cycles.

**Methods:**

One hundred infertile hyper-responder women who were candidate for IVF/ICSI were randomly assigned into two groups. Group 1 was given 20 mg Medroxyprogesterone acetate from day 1 of the menstrual cycle till trigger day. Group 2 was given GnRH antagonist (injection Cetrorelix 0.25 mg s/c) from the day when the leading follicle reached 14 mm until the day of trigger for the prevention of premature LH surge (flexible protocol). We measured LH serum levels on day 1, day 7 of cycle and on trigger day. The primary outcome measured was the incidence of premature LH surge. Other outcome measures were total number of mature follicles on trigger day, total number of mature oocytes retrieved and number of good quality day-3 embryos.

**Results:**

There was no premature luteinizing hormone surge in both groups of our study. The mean number of follicles on trigger day, mean number of M2 oocytes retrieved and mean number of good quality day-3 embryos were comparable in both the groups, with no statistically significant difference.

**Conclusions:**

The results of this study stated that MPA can be an effective alternative to GnRH antagonist for the prevention of premature LH surge in hyper-responder women undergoing COS for IVF. It is easy to use, widely available and cost-effective. It may establish a new regimen of ovarian stimulation using MPA as an oral alternative to GnRH antagonist treatment in hyper-responders.

## INTRODUCTION

Premature luteinizing hormone (LH) surge is one of the important causes for cycle cancellation during controlled ovarian stimulation (COS) in women undergoing IVF/ICSI cycles. LH secretion triggers ovulation in response to rapidly rising estradiol concentrations in a natural cycle, and premature LH surge can compromise oocyte yield in IVF/ICSI (Everett, 2006; Messinis, 2006). Efforts have been put forward to minimize the occurrence of premature LH surge. In order to avoid such an effect, pituitary suppression has been achieved using GnRH analogues over the last 40 years. Pituitary suppression was initially attempted using GnRH agonists in the 1980s, they bind to pituitary receptors in the hypophysis and induce the release of large amounts of FSH and LH (a flare-up effect) and an increase in the number of GnRH receptors (up-regulation) (Kumar & Sharma, 2014). However, after prolonged use, a GnRH agonist-receptor complex forms, which results in a decrease in the number of GnRH receptors (down regulation). As a result, the pituitary becomes refractory to stimulation by GnRH, leading to a decrease in circulating gonadotropins and thus preventing a premature LH surge. Though down-regulation by GnRH agonist promotes antral follicle synchronization, it requires daily administration of agonist and high doses of gonadotropins, as well as long treatment duration, which would be required to reach appropriate follicular development (Lambalk *et al.,* 2017). This leads to increased procedure complexity, higher cost and there is a risk of ovarian hyper-stimulation syndrome (OHSS), requiring HCG-trigger (Kuang *et al*., 2015).

GnRH antagonist can competitively inhibit endogenous GnRH and produce an immediate and rapid decline in LH and follicle-stimulating hormone (FSH) levels, without the flare effect of a GnRH agonist, and their administration by subcutaneous injection in the late follicular phase prevents an LH surge (Al-Inany *et al*., 2016; Bahçeci *et al*., 2005). However, we have seen that a varied proportion of patients (0.34%-38%) using GnRH antagonist protocol experienced premature LH surge, especially older patients and patients with diminished ovarian reserve (Bosch *et al*., 2003; Reichman *et al*., 2014). The antagonist protocol has been found to have fewer complications and to be more convenient for patients, because of the shorter treatment time and lower number of injections. But the antagonist protocol is expensive, and it also requires daily injections to be taken. Therefore, there is an unmet need for newer methods with comparable efficacy, safety profiles, cost effectiveness and more patient friendly.

Progestins can inhibit the pre-ovulatory LH surge when it is administered during the early part of the cycle, before estrogen priming. Progestin also alters pituitary responsiveness to GnRH and gonadotropin secretion. For more than 50 years, progestin has been widely applied to control ovulation in hormonal contraception (Heikinheimo *et al*., 1996; Evans *et al*., 2002) by blocking the LH surge; and since 2014 its use has been extended to prevent premature ovulation in IVF (Kuang *et al.,* 2015). In previous decades, progesterone could not be considered for use during ovarian stimulation, because of its negative impact on endometrial receptivity. Advances in embryo vitrification techniques with a post-warming survival rate very close to 99% has enabled clinicians to do frozen embryo transfers (La Marca & Capuzzo, 2019). It also enabled Reproduction Experts to consider new strategies for using progestin as an alternative to GnRH analogue for preventing premature surge in IVF, as vitrification removes possible harmful effects of progestins on endometrial receptivity. The transfer of cryopreserved-thawed embryos in the freeze-all embryo protocol has been reported to result in improved pregnancy and better delivery outcomes (Devroey *et al.,* 2011; Doody, 2014).

Medroxyprogesterone acetate (MPA) could be an alternative treatment in preventing early LH surge in patients undergoing IVF treatment, with the advantage of yielding a beneficial effect on serum LH, low-cost, easy use and availability. Need of embryo freezing and transfer in subsequent cycle is still an issue with this protocol. If equally efficacious, this protocol may find its place in oocyte donation/oocyte freezing cycles, where endometrial condition of the woman is not considered. Since the ‘freeze-all’ strategy with delayed transfer is preferable in hyper-responders, they could benefit more from this protocol.

## MATERIALS AND METHODS

After institutional ethics committee approval, one hundred infertile hyper-responder women who were candidates for IVF/ ICSI cycles at our IVF and Endoscopy center, from April 2021 to July 2021, were included in this study after signing an informed written consent form.

The inclusion criteria were as follows: age younger than 35 years, anti-Mullerian hormone (AMH) levels greater than equal to 3.5 ng/ml, weight less than 60 kg, presence of both ovaries and antral follicle count (AFC) >15 on day 1-3 of the menstrual cycle, and normal or near normal semen parameters. The exclusion criteria were age more than 35 years, AMH < 3.5 ng/ml, AFC <15 and severe male factor infertility.

The eligible women were allocated alternatively into one of the two groups: Group 1 (MPA + hMG; n=50) and Group 2 (GnRH antagonists + hMG; n=50). All patients were given oral contraceptives pills for 14 days as per our protocol. Group 1 was administered hMG (150-225 IU) and MPA (20 mg/d) from day 1 of cycle onward. Group 2 was administered hMG (150-225 IU) and injection of Cetrorelix 0.25 mg subcutaneously when the largest follicle reached 14 mm in size (flexible protocol). Follicular monitoring started on day-5 of the cycle and was performed every 2-4 days using transvaginal ultrasound examination to record the number of developing follicles. Serum LH concentrations were measured on day 1, day 7 and on trigger day for all patients. When the majority of the cohort reached above 17 mm in diameter, the final stage of oocyte maturation was triggered using a subcutaneous injection of decapeptyl 0.2 mg. We noted the total number of follicles (> 17 mm) on the day of trigger. Transvaginal ultrasound-guided oocyte retrieval was conducted 35 hours after trigger. Total number of mature M2 oocytes were noted in both groups. ICSI was performed on all M2 oocytes as per our standard protocol. The embryo cultures were performed according to standard procedures. The embryo morphology was scored according to the Atlas of Human Embryology (Magli *et al*., 2012). All good-quality embryos (including grade 1 and grade 2, 8-cell embryos) were frozen by vitrification on the third day after oocyte retrieval. The vitrification procedure for freezing cleavage-stage embryos was performed using the Cryotop carrier system.

### Outcome measurements

Primary outcome - The primary outcome is the incidence of premature LH surges, defined as serum LH >15 mIU/ml on the trigger day.

Secondary outcome measures, total number of mature follicles on the day of trigger, number of mature oocytes retrieved, number of good quality day-3 embryos.

### Statistical analysis

Data analysis performed by using the SPSS (Statistical package for Social Sciences) version 27:0. Qualitative data variables expressed by using frequency and percentage (%). Quantitative data variables expressed by using the Mean and the SD. The unpaired t-test was used to compare the quantitative data variables in both the groups. A *p*-value < 0.05 was considered significant.

**Ethics Approval**: We obtained the institutional ethics approval.

## RESULTS

### Patient characteristics

The average age of patients in Group 1 was 30.21, and in Group 2 it was 28.80, and there was no statistically significant difference between them (*p* value 0.135). The average weight of the patients in Group 1 was 58.47 and in Group 2 it was 61.48, and there were no significant differences between them (*p* value 0.257). Duration of infertility in Group 1 and Group 2 was 4.57 years and 4.4 years, respectively; and there was no significant difference between them (*p* value 0.86). The mean AMH levels in Group A was 5.422.65, and in Group B it was 7.01, and there was no statistically significant difference between them (*p* value 0.089). The mean AFC levels in Group A was 17.344.15, and in Group B it was 18.543.99; and there was no statistically significant difference between them (*p* value 0.143), as represented on Table 1.

**Table 1:** Patient characteristics.

Characteristics	Group 1 (n=50)	Group 2 (n=50)	*p* value
Age (years)	30.213.10	28.803.23	0.135
Weight (kg)	58.476.15	61.4811.97	0.257
Duration of infertility (years)	4.573.22	4.402.51	0.86
AMH (ng/ml)	5.422.65	7.013.75	0.089
AFC	17.344.15	18.543.99	0.143

### Cycle characteristics

**Figure 1:**
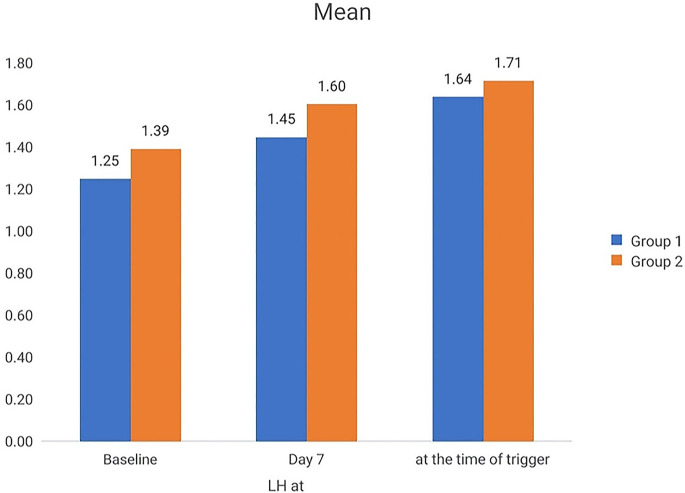
Luteinizing hormone levels.

There was no premature luteinizing hormone surge in both the groups in our study, as per shown in Table 2 and Figure 1.

**Table 2:** Luteinizing hormone levels.

LH at	Group 1 (n=50)		Group 2 (n=50)		p-value
	Mean	SD	Mean	SD	
Baseline	1.25	0.39	1.39	0.34	0.199
Day 7	1.45	0.40	1.60	0.29	0.152
at the time of trigger	1.64	0.37	1.71	0.30	0.452

*p*-value > 0.05 (Not Significant) Unpaired t-test used

The mean number of mature follicles on trigger day in the two groups were 18.39 and 20.30, respectively (*p* value 0.302). The mean number of M2 oocytes retrieved were 14.44 and 16.07, in the first and second groups, respectively (*p* value 0.092). The mean number of good-quality day-3 embryos in groups 1 and 2 were 12.61 and 13.41, respectively (*p* value 0.280); as shown in Table 3.

**Table 3:** Cycle characteristics.

	Group 1	Group 2	*p*-value
N	50	50	
Mature follicles on day of trigger (n)	18.39±5.26	20.30±9.86	0.302
M2 Oocytes retrieved (n)	14.44±3.99	16.07±5.47	0.092
Day 3 good quality embryos(n)	12.61±3.55	13.41±3.82	0.280

*p*-value (Not Significant). Unpaired t-test used

## DISCUSSION

Progestins have been found to prevent LH surges by blocking estradiol-induced GnRH surge. The results of this study support the hypothesis that MPA can be an effective alternative to GnRH antagonist for the prevention of premature LH surge in women undergoing COS for IVF. It is easy to use, widely available and cost-effective. Additionally, transferring the embryo in subsequent cycles could increase implantation and pregnancy rates (Wang *et al*., 2018). This paves the way for establishing a new regimen of ovarian stimulation using MPA as an oral alternative to GnRH analogue treatment in combination with oocyte or embryo cryopreservation.

In 2015, Kuang *et al*. indicated that MPA was effective in the prevention of premature LH surge in a woman who underwent controlled ovarian stimulation (Lambalk *et al.,* 2017). Similarly, there was no incidence of premature LH surge in our study. Wang *et al*. (2016) also concluded that there was no incidence of premature LH surge in both groups.

Progestin-induced pituitary suppression during controlled ovarian stimulation is still in the course of exploration. Wang *et al*. (2018) in a large retrospective cohort study, concluded that the neonatal outcomes and risk of congenital malformations were similar between the PPOS and the conventional GnRH-a short protocol. Birth characteristics, including gestational age, birth weight, infant sex and early neonatal death were also comparable between the two groups. The incidence of live-birth defects was similar in both groups (1.52% *vs*. 1.63%). Because of its effectiveness and safety, progestins have been accepted by IVF clinics worldwide, and have been widely used in patients defined as normal responders or poor responders, and in patients with PCOS since 2016, and showed optimal ovarian response and IVF outcomes (Zhu *et al*., 2017; Chen *et al*., 2017; Yucel *et al*., 2014).

Different types of synthetic progestins (dydrogesterone, utrogestin and MPA) effectively suppressed the premature LH surge and produced a comparable number of viable embryos and pregnancy outcomes (Zhu *et al*., 2017; Yu *et al*., 2018). In this study, we chose MPA as it did not interfere with the measurement of endogenous progesterone and seemed better than Dydrogesterone for suppressing LH surge (Yu *et al*., 2018). Richter *et al.* (2002) and Chabbert-Buffeta *et al.* (2000) indicated that progesterone prevents induced LH surge against E2 in early stages of signal transmission when started on day-3 of the cycle. But later administration, in mid follicular phase was not effective in preventing LH surge.

The results of the study indicated there was no significant difference between the number of mature follicles, mature M2 oocytes retrieved and good quality day-3 embryos in the two groups. In addition, medroxyprogesterone acetate had no negative effect on growth, development of oocytes and embryos. These results indicate that medroxyprogesterone was effective without any complications. These results agreed with previous studies by Kuang *et al*. (2015) and Wang *et al*. (2016). The number of expected oocytes were also in acceptance in both groups. One of the disadvantages with progestin treatment is that we cannot do fresh embryo transfers. Embryo freezing is mandatory, and then transfer in the subsequent cycle.

Therefore, MPA could be the first choice for ovarian stimulation in fertility preservation, oocyte donation and preimplantation genetic testing cycles. Non-conventional ovarian stimulation protocols (luteal and random-start, double ovarian stimulation), which always require oocyte or embryo cryopreservation, and it may also use progestins to inhibit endogenous LH surges.

The highest limitation of this study is the small patient population. Future studies with enlarged sample size are needed to generalize the findings from this study.

## CONCLUSION

The study shows that MPA has similar efficacy, as GnRH antagonists for prevention of LH surge in hyper-responders. It also has added benefit of producing good-quality oocytes and embryos as GnRH antagonists in hyper-responder patients undergoing COS. Medroxyprogesterone acetate could be used as an appropriate medication for suppressing LH levels in place of GnRH antagonists. In our study, we found that MPA is more economical, patient friendly with comparable effects as GnRH antagonist with no complications. Additional large multi-center randomized control trials are needed to compare live-birth rates in the two treatment modalities.
